# Therapy-refractory lymphatic fistulas following complete lymph node dissection in malignant melanoma: local radiotherapy as an effective therapeutic modality to avoid cancer treatment delay

**DOI:** 10.1007/s00423-026-03972-0

**Published:** 2026-01-21

**Authors:** Johannes Röttgen, Maximilian Coerper, Jonas Dohmen, Daniel Weissinger, Steffi Marx, Philipp Leyendecker, Judith Sirokay, Azin Jafari, Jennifer Landsberg, Jörg C. Kalff, Philipp Lingohr, Alexander Semaan

**Affiliations:** 1 Department of General, Visceral and Vascular Surgery, University Hospital Bonn, Venusberg-Campus 1, Bonn, 53127 Germany; 2 Center for Skin Diseases, Clinic for Dermatooncology and Phlebology, University Hospital Bonn, 53127 Bonn, Germany; 3 Department of General and Visceral Surgery, Charité Campus Benjamin Franklin, Hindenburgdamm 30, 12203 Berlin, Germany

**Keywords:** Malignant melanoma, Complete lymph node dissection, Lymphatic fistula, Radiotherapy, Cancer treatment delay, Therapy algorithm

## Abstract

**Purpose:**

Complete lymph node dissection (CLND) for stage III malignant melanoma is associated with substantial morbidity, particularly lymphatic fistulas (LF), which may delay adjuvant therapy. Grade C LF, characterized by prolonged secretion and clinical complications, lacks a standardized treatment algorithm. This study evaluated the efficacy and safety of radiotherapy in managing Grade C LF after axillary or inguinal CLND in malignant melanoma.

**Materials and methods:**

We conducted a retrospective, single-center study of patients with stage III malignant melanoma who developed Grade C LF after axillary or inguinal CLND between 2013 and 2021 at the University Hospital Bonn. Radiotherapy was administered as either low-dose or high-dose protocols. Treatment success was defined as LF resolution within 30 days post-radiation. Toxicities were assessed using RTOG and RTOG/EORTC scores.

**Results:**

Among 352 CLND procedures, 32 patients (9.1%) developed Grade C LF. Radiotherapy achieved resolution in 84.4% (27/32) of cases, independent site (inguinal vs. axillary, *p* = 0.999), laterality (*p* = 1.000), or timing of radiation initiation (*p* = 0.518). Low- and high-dose protocols achieved comparable success rates (89.5% vs. 76.9%, *p* = 0.347). However, high-dose radiotherapy caused significantly more acute (46.2% vs. 10.5%, *p* = 0.038) and long-term toxicities (38.5% vs. 5.3%, *p* = 0.029). Mean time to therapy success was 27 days after radiation initiation and 64 days after surgery, supporting adherence to the ESMO’s 12 week adjuvant window.

**Conclusion:**

Grade C LF occurs in approximately 10% of patients following CLND and can delay adjuvant therapy. Radiotherapy, particularly low-dose protocols, is a safe and effective strategy to resolve LF and minimize treatment delays.

## Introduction

Malignant melanoma represents the most aggressive and life-threatening form of skin cancer. Over the past five decades, its incidence has risen markedly worldwide, particularly among Caucasian populations, highlighting a growing public health concern with substantial global ramifications [1–3]. Various therapeutic modalities exist for addressing the different stages of malignant melanoma. According to the guidelines of the European Society for Medical oncology (ESMO), the primary treatment for malignant melanoma is surgical resection [4]. In cases of defined tumor thickness or other morphological characteristics, a sentinel lymph node biopsy (SLNB) should be performed. When lymphogenic metastasis without distant metastasis is present, as defined by the American Joint Committee on Cancer (AJCC) as stage III melanoma, a complete lymph node dissection (CLND) is recommended [4]. Common CLND regions include the groin and axilla as lymphatic drainage areas [5].

CLND is associated with a high risk of complications, including surgical side infections and other morbidities [6, 7]. One of the most prevalent complications is the formation of a lymphatic fistula (LF), resulting from trauma and damage to the lymphatic tissue [8].

The incidence of LF is a significant issue, often leading to further complications such as superinfections, prolonged hospital stay and cancer treatment delay [6, 9].

Although LFs are relatively common and therapeutically challenging, a universally accepted classification system for their severity is lacking, primarily due to limited comparability and inconsistent documentation in the literature.

Gerken et al. proposed a grading system for LFs following CLND in the inguinal or axillary region [10].

Since no single effective therapy for LF has been established, a range of conservative and surgical treatments is available. These including the administration of locally effective agents, such as fibrin glue, talcum, erythromycin or doxycycline, as well as surgical intervention like fistula ligation or a vacuum sealing therapy [8, 11–15]. Local radiation therapy is an alternative option described as an efficient modality for the rapid and effective obliteration of LFs, especially after vascular surgery where LFs are also frequent [5, 16–18].

In the present study, we evaluated the treatment success of radiation therapy for persistent Grade C LF following axillary and inguinal CLND in patients with lymphatic metastases of malignant melanomas.

## Methods

### Study design

From 2013 to 2021, a retrospective, single-center study was conducted at the University Hospital of Bonn, Department of Surgery, to investigate lymphatic fistulas following complete inguinal or axillary lymph node dissection. The indication for these procedures, in accordance with ESMO guidelines, was a resectable stage III melanoma [4]. According to the local standard operating procedure, all visible lymphatic vessels were ligated, followed by coagulation of persistent blood and lymphatic vessels and a subcutaneous wound adaption. Prior to the surgical wound closure, a suction drain was placed in each case of CLND. Postoperatively, patients received compression therapy consisting of initial local wound pressure and the use of compression stockings on the affected limb.

### Definition and grading of LF

In accordance with the definitions proposed by Ly et al. and Gerken et al., LF was defined as the leakage of lymphatic fluids following the injury of lymphatic vessels during surgical procedure [8, 10].

We adopted grading system suggested by these authors, which defines LF as a persistent secretion exceeding 50 ml within 24 h, considering the postoperative time interval and associated complications. The grading criteria are summarized in Table 1. In brief, higher LF grades correspond to prolonged duration of leakage, wound infections, the necessity for (surgical) interventions, and delays in treatment [10] (Table 1). In the present study, we focused exclusively on the treatment of Grade C LF following CLND.

**Table 1:** Proposed and adapted grading of LF after RLND by gerken et al. [10]

Grade	Definition
A	Persistent lymphatic leakage > 5 postoperative days, < 10 postoperative days. Absence of other wound complications
B	Persistent lymphatic leakage ≥ 10 postoperative days or lymphoceles requiring interventions
C	Lymphatic leakage leading to reoperation or subsequent conflict with medical measures or return to normal life
0	No lymphocele

Definition of lymphatic leakage, proposed by Gerken et al.:

Persistent secretion of lymphatic fluid (≥ 50 ml/24 h) from the surgically inserted drains or from the wound for more than 5 days or, after drainage removal, postoperative fluid accumulation within the cavity of resection provided the absence of wound dehiscence (lymphocele, lymphocyst, seroma) limiting the adherence of the wound surface.

### Treatment success of grade C lymphatic fistula

Treatment success was defined as the complete resolution of lymphatic drainage without the need for further intervention, specifically characterized by the absence of secretion within 30 days following the completion of radiotherapy.

For the evaluation of radiation-induced side effects, a distinction was made between short-term and long-term effects. The RTOG acute radiation morbidity scoring criteria and the RTOG/EORTC late radiation morbidity scoring scheme were employed for this differentiation [19]. An adapted summary of the scoring systems for skin and subcutaneous tissue as affected organs in radiotherapy of the axillary and inguinal regions is presented in Table 2.

**Table 2:** Adopted grading of acute (RTOG criteria) and long-term (RTOG/EORTC criteria) radiation-induced toxicities for skin and subcutaneous tissue in axillary and inguinal radiotherapy for lymphatic fistula following CLND [19]

Grade	Skin (acute)	Skin (late)	Subcutaneous tissue (late)
0	None	None	None
1	• Follicular faint • dull erythema • epilation • dry desquamation • decreased sweating	• Slightly atrophy • Pigmentation change • some hair loss	• Slight induration (fibrosis) • loss of subcutaneous fat
2	• Tender/bright erythema • Patchy, moist desquamation • moderate edmea	• Pathy atrophy • moderate telangiectasia • total hair loss	• Moderate asymptomatic fibrosis • slight field contracture < 10% linear reduction
3	• Confluent, moist desquamation other than skin folds • pitting edema	• Marked atrophy • gross telangiectasia	• Severe induration • loss of subcutaneous tissue • field contracture > 10% linear measurement
4	• Ulceration • Hemorrhage • Necrosis	• Ulceration	• Necrosis
5	Death directly related to radiation effects		

### Data acquisition and statistical analysis

Statistical analyses were performed using SPSS^®^ (IBM). Descriptive statistics included the calculation of absolute and relative frequencies, as well as the range, mean and median values. Statistical significance for categorial variables was assessed using Fisher’s exact test, while univariate binary logistic regression was applied for continuous variables. A p-value less than 0.05 was considered statistically significant.

## Results

### Patient cohort and therapeutic results

**Fig. 1:**
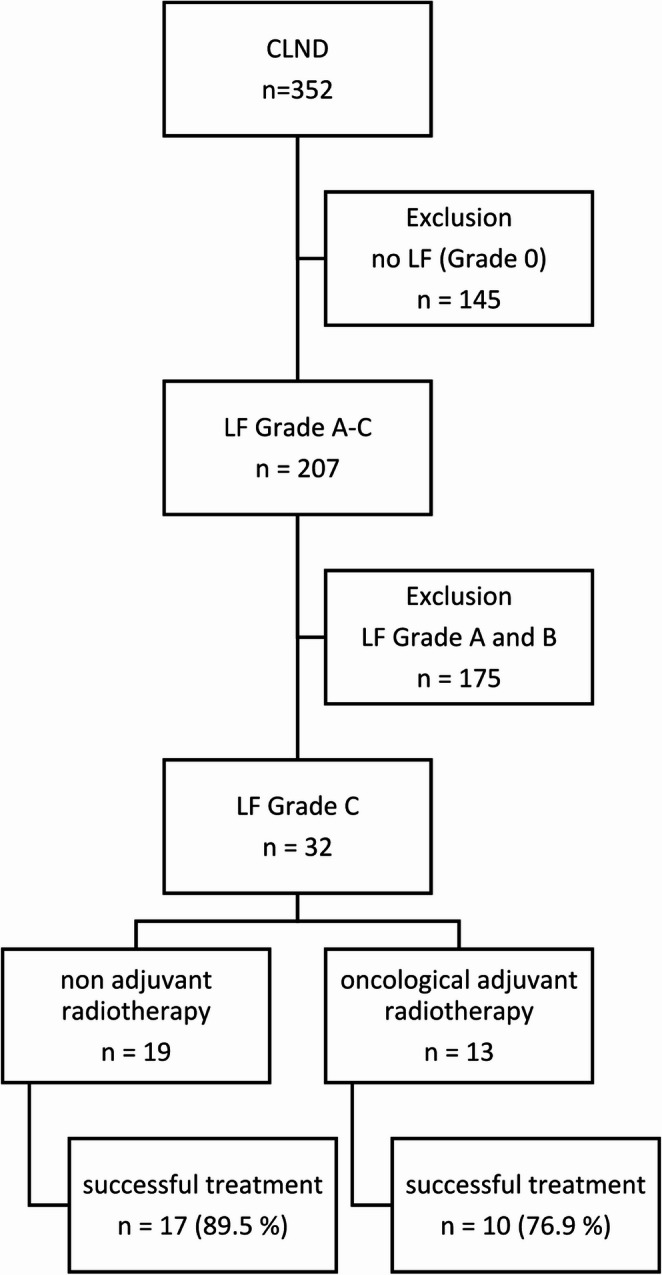
Flow chart of Patient Enrollment for Radiotherapy of LF after CLND

From 2005 to 2021, a total of 352 complete inguinal or axillary lymph node dissections were carried out at the University Hospital of Bonn, Department of Surgery. The patient cohort included in this study is illustrated in the flow chart presented in Fig. 1.

Postoperative complications, graded according to the widely used Clavien-Dindo Classification, occurred a rate of 23,2% for Grade III or higher complications (Table 3) [20].

**Table 3:** Clavien-Dindo classification (CDC) - Postoperative complications after CLND

CDC Grade	Total cohort
	n=352
0	120 (34,1%)
I	122 (34, %)
II	26 (7,4%)
III	82 (23,3%)
IIIa	34 (9,7%)
IIIb	48 (13,6%)
IV	2 (0,6%)

### Radiotherapy as a potential treatment for grade C lymphatic fistula

Despite the implementation of the described preventive measures, postoperative Grade C lymphatic fistula occurred in 9% (*n* = 32) of the investigated CLND procedures. Of these, 62.5% (*n* = 20) were located in the inguinal region, and 37.5% (*n* = 12) in the axillary region. All 32 Grade C fistulas were treated with local radiotherapy, which proved effective in 84.4% (*n* = 27) of cases. In four patients, prolonged drainage therapy or repeated punctures were necessary for more than 30 days following the completion of radiotherapy. In one case, lymphatic vascular sclerotherapy was successfully performed.

Radiotherapy was administered with an implanted suction drain in 88% of the cases. In the remaining 12%, lymphatic output was managed either by spontaneous drainage or repeated punctures.

This study investigated potential factors influencing the efficacy of radiotherapy in treating Grade C LF. No significant difference in treatment success was observed between anatomical regions; radiotherapy was similarly effective in both inguinal and axillary LFs (*p* = 0.13). Likewise, the laterality (left vs. right side) had no impact on treatment success (*p* = 1.00).

The mean time to initiation of radiotherapy was 29 days after the initial surgical procedure and showed no statistically significant association with treatment success (*p* = 0,518).

In the investigated cohort, treatment success was achieved on mean 27 days (range: 6 to 520 days) after the start of radiotherapy and 64 days (range: 23 to 590 days) after initial surgery. A detailed overview of patient characteristics and statistical findings is presented in Table 4.

**Table 4:** Patients characteristics

Cohort of Grade C LFn=32	n (%) / Median / Mean (range)	p-value (therapy success)
Sex (male: female)	21:11	0,637^§^
Age (years)	67^md^ (39-83)	0,988^#^
Postoperative LF (total) None Grade A and B Grade C	207 (58,8 %) 145 (41,2 %) 175 (49,7 %) 32 (9,1 %)	
Localization of LF axillary inguinal Laterality left right	12 (37,5 %) 20 (62,5 %) 18 (56,3 %) 14 (43,7 %)	0,999^§^ 1,000^§^
Initiation of radiation therapy (POD)	29^m^ (7-76)	0,518^#^
Therapy-success after initiation of radiation(days)	27^m^ (6-520)	
Therapy-success after initial Operation (POD)	64^m^ (23-590)	

md = median, # = binary logistic regression, m = mean, § = fisher exact test

To further investigate potential factors influencing treatment success, we retrospectively analyzed the two distinct radiation protocols applied in our study. In 59.4% of cases (*n* = 19), a non-oncological low dose radiotherapy aimed explicitly at eradicating Grade C LF was administered. For this purpose, the total irradiation dose ranged from 6 to 20 Gy.

The remaining 40.6% (*n* = 13) of patients received adjuvant oncological radiotherapy with a total dose of 50–60 Gy. The primary objective of this protocol was the treatment of the locally advanced malignancy, with fistula resolution as a secondary goal.

Retrospective analysis demonstrated that both adjuvant and non-adjuvant radiation protocols were similarly effective in treating Grade C LF (*p* = 0,245). Detailed information on the protocols and statistical outcomes is presented in Table 5.

**Table 5:** Adjuvant vs. non adjuvant radiation protocols

	total cohort *n* = 32	non adjuvant radiation *n* = 19	adjuvant oncological radiation *n* = 13	*p*-value
Therapy success (%)	27 (84,4%)	*n* = 17 (89.5%)	*n* = 10 (76.9%)	0.347^§^
Irradiation dose (single dose/total dose)		1–1.8 Gy/6–20 Gy	2–2.4 Gy/50–60 Gy	0.347^§^
Short term side effects (RTOG) Grade 1 Grade 2 Grade 3	8 (25%) 7 (87.5%) 0 (0%) 1 (12.5%)	2 (10.5%)	6 (46.2%)	0.038^§^
Long term side effects (RTOG/EORTC) Grade 1 others	6 (18.8%) 6 (100%) 0 (0%)	1 (5.3%)	5 (38.5%)	0.029^§^

§ = fisher exact test

### Adjuvant oncological radiotherapy: elevated risk of short- and long-term toxicities

Acute radiation-associated toxicity was observed in eight patients (25%) of the cohort. Seven cases were classified as Grade 1 adverse event according to the RTOG criteria, while one Patient experienced a Grade 3 toxicity. Two patients (6.25%) discontinued treatment due to adverse events.

Following completion of the radiotherapy, six patients (18.8%) developed a Grade 1 long-term toxicity, as assessed by the RTOG/EORTC late radiation morbidity scoring system.

Although adjuvant radiation proved to be an effective treatment modality for Grade C LF, it was associated with significantly higher rates of both acute (*p* = 0.038) and long-term (*p* = 0.029) radiation-related toxicities.

## Discussion

Lymphatic fistulas after CLND are frequent and challenging complications. Grade C LF is of particular concern due to its potential to delay cancer treatment, which represents the most critical aspect of LF following CLND in malignant melanoma [10]. According to ESMO guidelines, adjuvant treatment should be initiated within 12 weeks after CLND [4].

Several studies have investigated radiation therapy for the treatment of lymphatic fistula after vascular surgery; however, to our knowledge, no series have evaluated radiation therapy for Grad C LF after CLND [21, 22].

In the present study, we observed a mean treatment success of radiotherapy at 27 days after initiation of therapy and 64 days after the initial surgical procedure. This timeline aligns with guideline-compliant adjuvant treatment and represents an effective strategy to avoid cancer treatment delays in cases of Grade C LF, which occurs in approximately 10% of patients following CLND.

Given that our study found no significant effect of the timing of therapy initiation on treatment success, early initiation is recommended to ensure adherence to guideline-based adjuvant therapy [4]. Similarly, Hautmann et al. investigated the efficacy of radiation therapy for LF following vascular surgery and found no difference in treatment outcomes between whether radiotherapy was initiated before or after the first 10 postoperative days [18].

In the available literature on radiotherapy of LF following vascular surgery, the sole indication for irradiation was fistula management. Total radiation doses reported in these studies varied widely, ranging from 3 Gy to 18 Gy [21, 22].

In contrast, our study includes both low-dose radiation treatments and oncological adjuvant irradiation. Current literature reports success rates ranging from 78% to 100% for low-dose therapy in treatment of LF following vascular surgery, which are consistent with the outcomes observed in our study [21, 22]. We found that low-dose radiation is equally effective as oncological adjuvant radiation in treating LF after CLND. In cases where adjuvant radiation is already planned, the treatment of lymphatic fistula can be performed concurrently.

As expected, our study revealed a significant higher risk for radiation-related side effects with increasing total irradiation dose. If non-oncological irradiation is recommended, we suggest maintaining the radiation dose for LF obliteration as low as possible, with effective doses in our study ranging from 6 to 20 Gy in total.

**Fig. 2:**
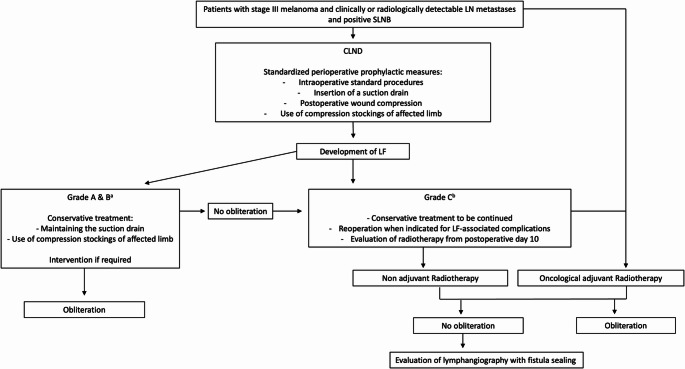
Therapy algorithm for LF after CLND

Based on our results, local radiation therapy appears to be a safe and effective option for managing Grade C LF following CLND in malignant melanoma. The currently center-specific therapeutic algorithm is illustrated in Fig. 2. Based on our findings and those of the cited vascular surgery literature, we suggest that the algorithm may serve as a general management framework for axillary or inguinal lymphatic fistula following CLND.

As alternative, non-operative treatment for LF in the axillary and groin regions, local application of fibrin glue, talcum, erythromycin and doxycycline has been described [11–13, 15]. However, only a limited number of studies, mostly small case series or case reports, are available. While these reports demonstrate a 100% successrate, they are subject to considerable bias due to the small cohort size, with no more than five patients per study [11–13]. In contrast, local radiation therapy for LF is a more studied and reliable treatment modality, particularly in the context of post-vascular surgery care [17, 18, 21]. Moreover, radiation therapy offers a non-invasive method, where the application of effective agents involves invasive procedures associated with a higher risk of complications [11–13].

Another interventional non-surgical treatment modality is lymphangiography with fistula sealing [23, 24]. According to the literature, success rates range from 77.8% to 80% [23, 25, 26]. Compared to our study lower rates of success are found with lymphangiography [17, 18, 21–23]. This technique is further limited by its technical difficulty, high costs, low reproducibility, and increased risk of procedure-related complications in interventional settings.

### Limitations

The present study has several limitations. Although it includes the largest cohort to date investigating radiation therapy for LF after CLND in malignant melanoma, the study is limited by its retrospective, single center design. Due to those structural limitations, including the overall small cohort size, we were unable to determine which radiation protocol was more effective for the treatment of Grade C LF following CLND.

## Conclusion

Approximately one in eleven patients develop a Grade C Lymphatic fistula after CLND in malignant melanoma. This complication may lead to a cancer treatment delay, which can impact the oncological outcome. According to ESMO guidelines, adjuvant treatment should be initiated no later than 12 weeks post- surgery. Our study demonstrates that radiation therapy of Grade C LF can successfully prevent a cancer treatment delay, even with low dose radiation. Furthermore, the efficacy of a radiation therapy is not influenced by the timing of therapy initiation, the intention of the radiation (adjuvant vs. non adjuvant), nor the anatomical location of the LF. Regarding adjuvant therapy, we recommend early initiation of radiotherapy to minimize any delay in subsequent oncological treatment.

Given the elevated risk of radiation-related side effects associated with adjuvant oncological radiation protocols when used solely for LF obliteration, low-dose radiation should be considered the preferred therapeutic modality.

## Data Availability

Data will be made available on request.
